# The Indonesian general practitioners’ perspectives on formal postgraduate training in primary care

**DOI:** 10.1186/s12930-018-0047-9

**Published:** 2018-11-13

**Authors:** Fitriana Murriya Ekawati, Mora Claramita, Wahyudi Istiono, Hari Kusnanto, Adi Heru Sutomo

**Affiliations:** 1 Department of Family, Community Medicine and Bioethics, Faculty of Medicine, Public Health and Nursing, Universitas Gadjah Mada, Radioputro 1st Floor, Jalan Farmako Sekip Utara, Sleman, Yogyakarta, Indonesia; 2 Department of General Practice, University of Melbourne, Melbourne, VIC Australia; 3 Department of Medical Education, Faculty of Medicine, Public Health and Nursing, Universitas Gadjah Mada, Yogyakarta, Indonesia

**Keywords:** Primary care doctors, Training, Family medicine, Indonesia, Qualitative study

## Abstract

**Background:**

To be recognized as a primary care physician (PCP), an Indonesian general practitioner (GP) has to follow a formal postgraduate training in primary care. However, 4 years since the regulation was published, the progress of the training is slow. There is a need to deeply investigate the doctors’ perspectives, particularly to explore factors associated with their willingness to follow this training.

**Aim:**

This study aims to explore the GPs’ views and perspectives related to the formal postgraduate training in primary care that may influence their enrolment in PCP program.

**Methods:**

We conducted semi-structured interviews with a topic guide. The study took place in Yogyakarta from January to December 2016. The participants were GPs practicing in Yogyakarta primary care clinics who were recruited using purposive-maximum variation sample design. The interviews were audio-recorded and transcribed. The data were analysed using interpretative phenomenological analysis approach.

**Results:**

Nineteen GPs participants were involved in this study. Three major themes were identified, namely unfamiliarity, resistance, and positivism. Almost all the GP participants were unfamiliar with the primary care training program. They were also pessimistic if the training could change the health service in the country while it lacked resources and infrastructures. However, exposure to the training brought positive insights that it could improve the doctors’ knowledge and skills in primary care practice.

**Discussion:**

The government intention to establish PCP training is currently on the right tract. However, information dissemination and more supports in primary care are also essential.

## Background

The World Health Organization (WHO) at the 30th commemoration of the Alma Ata Declaration in 2008 urged its country members to rethink their primary care services [1]. The narrative promises of this setting were broadly explained, calling for high-quality care for all. In 2005, the WHO declared the essential need of universal coverage for all people in the world, to help them afford essential health care without any financial barriers [2]. Then, in 2008, the WHO report emphasized that primary care improvement consisted of four major pillars: the health financing reform (universal coverage), health service improvement, leadership, and policy reform [1].

In response to the above WHO suggestions, the Indonesian government has been implementing the universal health coverage policy-which is also known as *Jaminan Kesehatan Nasional* (JKN). This program was launched in January 2014 and was designed to expand its broad coverage until 2019 [3, 4]. In line with the JKN implementation, the Indonesian government had also prepared a structured formal postgraduate training for general practitioners (GPs) to upgrade their skills in primary care. The training was stipulated in Medical Education Act number 20-year 2013 [5]. In this regulation, the terminology of ‘primary care physician’ (PCP) was introduced as a doctor having an equivalent position as a specialist. To be recognized as a PCP doctor, the GPs have to follow a formal postgraduate training in primary care conducted by 17 medical faculties licensed by the Indonesian Ministry of Education. The PCP curriculum content was designed according to international recommendations of family medicine training, consisting of advanced training for the GPs to have more person-centred care, continuity of care, community-oriented, holistic and comprehensive care for their patients, leadership in general practice, and primary care management [6, 7].

The formal primary care doctor training for Indonesian doctors is arranged with a workplace-based learning methods (WBL) [8] developed by the Indonesian National Board of PCP (the representative members of the 17 medical faculties). The WBL scheme aimed to specifically train the future doctors to reflect and learn medical cases from their practice settings. There are two sub-schemes in WBL methods: the first scheme is designed for those physicians who already graduated from the university and have had at minimum of 5-years practice in primary care. This mechanism is also known as the “Recognition Prior Learning” (RPL) model, in which the doctors only need to accumulate credits by taking a 6-month PCP training at assigned universities. The RPL will be completed by 2030 to provide adequate opportunities for the current GPs to join this scheme. The second scheme of WBL is the regular 2–3 years training scheme, which is designed for newly graduate GPs with less than 5 years practice experience. To do the training, the junior GPs need to be registered as PCP residents at a licenced university.

Unfortunately, until 4 years since the medical education act was published, the formal postgraduate training in primary care was not fully established. There had been strong national debates related to this law from the Indonesian Doctors Association [9, 10]. A juridical review was proposed by the Indonesian Medical Association and claiming that the practice skills had actually been included in undergraduate medical curriculum. Subsequently, the association notified the government to take more consideration before the training was launched nationally [9]. The Indonesian medical education experts constrained this claim by stating that the essence of primary care practice was different from the undergraduate degree. They added that the PCP training would help doctors to improve their skills [11–13] and would equip them with an advanced focused clinical professional development (CPD) in primary care practice [14].

Until this publication was written, very limited evidence was available to explore the Indonesian doctors’ perspectives about this formal postgraduate training. The local investigation about the GPs views related to the training was insufficient and were dominantly from news or media releases [15, 16]. Being intrigued by the debates, this study sought to deeply investigate the Indonesian GPs’ views on this training, specifically, what are their arguments to follow or not to follow the government intention to upgrade their knowledge and skills in primary care.

## Methods

This research applied a phenomenology approach to develop a comprehensive understanding of the GP participants’ views [17]. Consistent with this methodology, the data collection was conducted using semi-structured interviews to provide greater opportunities for the participants to express their views.

This study took place in Yogyakarta from January to December 2016. A purposive maximum variation sampling strategy was applied to ensure adequate encapsulation of the participants’ perspectives. The GPs recruitment process was done in both private and public primary care clinics from five Yogyakarta regions (Kulonprogo, Sleman, Yogyakarta city, Bantul, and Gunung Kidul). The recruitment process is described as follows: FME (first author) advertised this study in Yogyakarta general practitioners’ networks and mailing lists-with more than 150 GPs members. The GPs who were interested to join the interview, were advised to contact FME, and FME then contacted back the GPs personally for the interview scheduling. Written informed consent was obtained from all GP participants, including their consent that this research would be published in a journal board using their anonymous identity. All the interviews were held in a private room in the GP’s clinics. All the interviews were audiotape-recorded. Each participant was given a small souvenir bag as a token of their participation.

All the interviews were based on a topic guide. At the beginning the GP participants were explained about the aims and any related information regarding this study. Then, three main guiding questions were asked to them: (1) their general views related the formal PCP training, (2) their concerns related to the PCP training and (3) their expectation for the training. Prompts were also applied to explore more on the GPs views, such as: silent pause, and minimum engagement, (i.e. *‘what do you think…’, ‘tell me more…’*) [12].

The interviews were transcribed and analyzed using Interpretative Phenomenological Analysis (IPA) approach for moderate scale participants. It is an analytical method to deeply understand the participants’ views combined with the researcher analysis behind the participants’ quotes. The steps of IPA analysis were systematically applied as had been suggested by Smith and Osborn [18]: (1) First, FME and MC (Co-author) read all the transcribed texts independently, to be familiar with the participants’ views. (2) Notable quotes were noted and discussed in three separate meetings. (3) The quotes were then grouped into themes and superordinate themes. Finally, (4) the emerging themes were discussed and crosschecked amongst the other co-authors with primary care backgrounds [12, 18].

## Results

Nineteen GPs were recruited. Most them were females, practicing in rural areas, aged 30–40 years, and having more than 5 years’ experience practicing as a GP in primary care practices. The participants had an equal proportion of GPs who practiced in Puskesmas (Indonesian Public Primary Care Clinics) and those who ran private practices. Ten GPs did not have any previous exposure to family medicine/primary care focused trainings, while nine GPs in this study had exposure to family medicine/primary care courses. Eight of the participants had joined the Universitas Gadjah Mada’s (UGM University-Indonesia) *Weekly Clinical Updates*^®^-family medicine postgraduate course for 1 year, and one GP participant was a Master of Family Medicine graduate from the university. The details of the participants are presented in Tables 1 and 2.

**Table 1:** Maximum variation sample details

Characteristic	Number
Gender	
Women	13
Men	6
Age	
25–40	13
41–55	4
56–70	2
Practice setting	
Rural	14
Urban	5
Practice type	
Public	9
Private	10
Practice duration	
Less than 5 years	4
5–10 years	7
10–15 years	3
> 15 years	5
Practice location	
Kotamadya	3
Sleman	6
Bantul	3
Gunungkidul	4
Kulonprogo	3

**Table 2:** Characteristic of each participant

Name	Practice setting	Practice duration	Experience of following a primary care/family medicine course(s)
Doctor 1	Urban	Less than 5 years	No
Doctor 2	Rural	5–10 years	No
Doctor 3	Rural	> 15 years	No
Doctor 4	Urban	5–10 years	No
Doctor 5	Urban	5–10 years	No
Doctor 6	Urban	Less than 5 years	No
Doctor 7	Rural	> 15 years	Yes (1 year)
Doctor 8	Rural	> 15 years	Yes (1 year)
Doctor 9	Rural	5–10 years	Yes (1 year)
Doctor 10	Rural	> 15 years	Yes (1 year)
Doctor 11	Rural	> 15 years	Yes (1 year)
Doctor 12	Rural	10–15 years	No
Doctor 13	Rural	10–15 years	Yes (1 year)
Doctor 14	Rural	10–15 years	Yes (MSc in Family Medicine)
Doctor 15	Rural	Less than 5 years	No
Doctor 16	Rural	Less than 5 years	Yes (1 year)
Doctor 17	Urban	5–10 years	Yes (1 year)
Doctor 18	Rural	5–10 years	No
Doctor 19	Rural	5–10 years	No

The interviews went well. FME had no difficulties to have the interviews with the participants. All of them had also been informed that their participation would not affect their relationships with the FME or other co-authors’ institution, now or in the future [12].

Three superordinate themes were identified as unfamiliarity, resistance, and positivism. Unfamiliarity referred to the doctors who were unfamiliar with the PCP program: about what it was, for whom, and if they would have any compensation related to the training they would have in the future. Resistance reflected the participants’ perspectives and pessimistic thought that the training could improve the health system. However, some physicians who had previous primary care training acknowledged that this training benefited their knowledge and practice skills in primary care.

### Unfamiliarity

Almost all doctors in this study expressed their unfamiliarity related to the PCP terminology. They were unclear about: who were the PCPs? and What made the PCP doctors different from current general practitioners, as both kinds of physicians would work in the same clinics. The doctors were also unsure if they would have a different recognition in front of their patients, as what had been said by Doctor 6: *“How is the position of the PCP doctors? Where are their positions? Would they practice in the different clinic than us in Puskesmas? Do we treat different patients?*” (Doctor 6, urban practice).

While it was stipulated in the regulation that to become primary care doctors the GPs should take a formal postgraduate training, the doctors were unsure about the clarity of the training. They questioned if the training was compulsory or a voluntary; and whether it would require them to leave their practice and take an on-campus training as similar to other specialist trainings. Unfortunately, with those questions, the doctors felt that there was limited information available for them. Without adequate explanation and clarification, their unfamiliarity resulted in doubts to go into training.

“*So, for the future of the training, I do not know about the length of the training. Do we have to take a 6*-*month training or is this just an alternative? Do we have to also leave our practice? I want to know about this. Otherwise, I could not decide what is the best for myself*” (Doctor 13, rural practice)

### Resistance

After expressing their unfamiliarity with the PCP program, half of the GP participants expressed their resistance to take the postgraduate training program. This was particularly expressed by doctors who felt that they were competent enough already. They thought that they had mastered and had given their best performance, referring to the list of GPs’ competencies, such as health promotion and prevention. They thought that there were no further additional skills needed for primary care doctors; as what had been said by Doctor 9: “*We had given our best performance in primary care, in our undergraduate curriculum we have been trained with the skills as a GP well. We had done the comprehensive care; we did the health promotion and prevention, we had also expressed our objection to this training” (Doctor 9, rural practice).*

These doctors also felt resistance and being pessimistic that their efforts to follow the PCP program would improve their practice because of the limited resources available in primary care. The doctors argued that their undergraduate skills were already adequate to tackle the patients’ problems, but the facilities prevented them to perform well. They thought that the training would not result in any improvement unless the government improves the facilities.

*“I may not be good at patient care. However, I think this is the best I can do. We already have adequate skills as GPs [from the undergraduate education]. But, look, why couldn’t we treat the hypertensive patients well? [because] We only have captopril in our clinic. I also agree that maybe half our patients are psychosomatic patients; we need a long time to gain that information. We do not have time to do that. Even for the doctors; we do not have enough doctors here. Only one doctor practicing in the clinic, another doctor went to a meeting to health office”* (Doctor 2, rural practice)

In addition, the GPs thought that the training would burden their economics situation. They argued that their undergraduate training in medical school was long enough to get good salaries compared to engineers or businessmen training. Furthermore, there were no such of a registrar’s salary in Indonesian medical training.

*“I am afraid that it would delay the time for the future GPs to be able to practice. You know that so far, we needed to finish our 4* *years training in an undergraduate degree, after that we continued to another 2* *years of clinical rotation. Not enough, we also had 1* *year of the internship program. Now we have to have another training to be able to practice? I cannot imagine. In another country, they have salaries for the residents, but here we pay the residency with our money. See that engineers, businessmen they only need 4* *years to work, we would need 8* *years to practice, that is too long…why don’t we just have a short course on this?”* (Doctor 15, rural practice).

For this question, interestingly, when the GPs were given a prompt to some GPs if she got a chance to follow another specialization training, with the similar situation of no payment for the residents, the doctors replied with “*Yes, I am interested. I want to pursue a specialist training, that is my dream. I adore this specialization since I was in Med*-*school*” (Doctor 19, rural practice).

This study also found an interesting argument that the continuation of PCP program was constrained by groups of doctors in the country. One GP commented this situation that there were doctors who did not support this PCP program exist.

*“This is interesting, I know that this program is disrupted by other doctors, this is ridiculous. Some doctors didn’t like their colleagues to upgrade their skills. I also knew that some doctors who constrained this training were actually not practicing in primary care, what are their problems? Some people in the Association also strongly debate this program. I think we just need to keep this going’ (Doctor 11, rural practice).*

### Positivism

Different perspectives were expressed by half of the doctors who already had more exposure to any family medicine trainings. Doctors who practiced in the rural areas expressed their gratitude as they already missed any training in general practice and believed that the training could improve their practice in primary care, as suggested by Doctor 19: *“I am happy with the training. So far, I’ve been practicing in rural areas, this is superb to remind us of the skills in primary care”* (Doctor 19, rural practice)

Some doctors told us more practical aspects of their course experience helped them to appropriately manage their patients in primary care. A doctor expressed his experience that his prescription to chronic disease patients changed significantly after he took his training in family medicine. After taking the training, he felt that he had another perspective on the patients’ care. Lately, he considered more aspects in his patient care.

*“I felt significant changes in myself as a doctor, previously I only met my hypertensive patients and gave the pills. I did not care about anything else. However, now, I feel that they need more my attention, I feel that I am responsible for their continuing medication, I should know that they have a risk of complications, stroke, heart attack, which may burden their lives and families. I am feeling like a real doctor, who not only need trust from the JKN insurance but more importantly from my patients and to manage their conditions”* (Doctor 14, rural practice).

Another GP in this study also promoted that the training could help the GPs’ practices during the implementation of JKN as Indonesian Universal Health Coverage (JKN). He wondered if the GPs could perform well in the clinic, the unnecessary referrals could be avoided and the patients would receive a more appropriate care.

*“I think the training would be more beneficial for our graduate doctors. I experienced it in my clinic. There are some newly graduate doctors working with us and to be honest, they did not have a similar experience as I do. They loved to refer patients to secondary care, that may be right, but I have a different opinion, this might also lead to unnecessary treatment to the hospital. What I expect is they evaluate the patients first, knowing their needs and treat them comprehensively”* (Doctor 11, rural practice)

## Discussion

This study has identified interesting perspectives from the Indonesian GPs about the PCP training that the clarity of the PCP information influenced the doctors’ views towards the training. It was mentioned that the doctors still questioned about the details of the training: such as its contents, length, the different authorities of PCP doctors and their future incentives compared to the current GPs. In addition, the practice supports and the medical association debates also challenged the doctors to opt in the training. Regarding this finding, the Indonesian government had actually disseminated information related to primary care doctors’ training. The Frequently Asked Questions (FAQ), the training schemes, the PCP competencies had been published [19]. Some scholarships to support the training were also offered [7]. However, seemed that the distribution and the clarity of those information inadequately reached the primary care doctors.

It also appeared at the result section (Resistance) that the doctors perspectives were framed with their current knowledge of general practice and accused its development by comparing the training with the facilities limitation in practice settings [14]. The views that the doctors were admiring the other specialist trainings might also show that they lacked abstraction and doubt the prospects of PCP graduates compared to other specialization. Moreover, the medical association did not fully support the training and published their objection [9].

Interestingly, it is showed that exposure to a formal training could be a powerful strategy to change the doctors’ perspectives about the primary care practice. Half of the participants who already followed the formal training in family medicine had better insights of the training compared to the other doctors who didn’t not have any exposures to family medicine courses. The doctors expressed that the training improved their practice perspectives, from treating the acute care to a more comprehensive and holistic care. From a disease-based treatment to a more personal care for the patients, and how to finally see the primary care as a macro system on its relationship with secondary care [20, 21]. While the formal training seem to be suspended because of the national medical association resistance, facilities improvement and supports for the trained doctors (such as: additional incentives) could change the situation [22, 23].

**Fig. 1:**
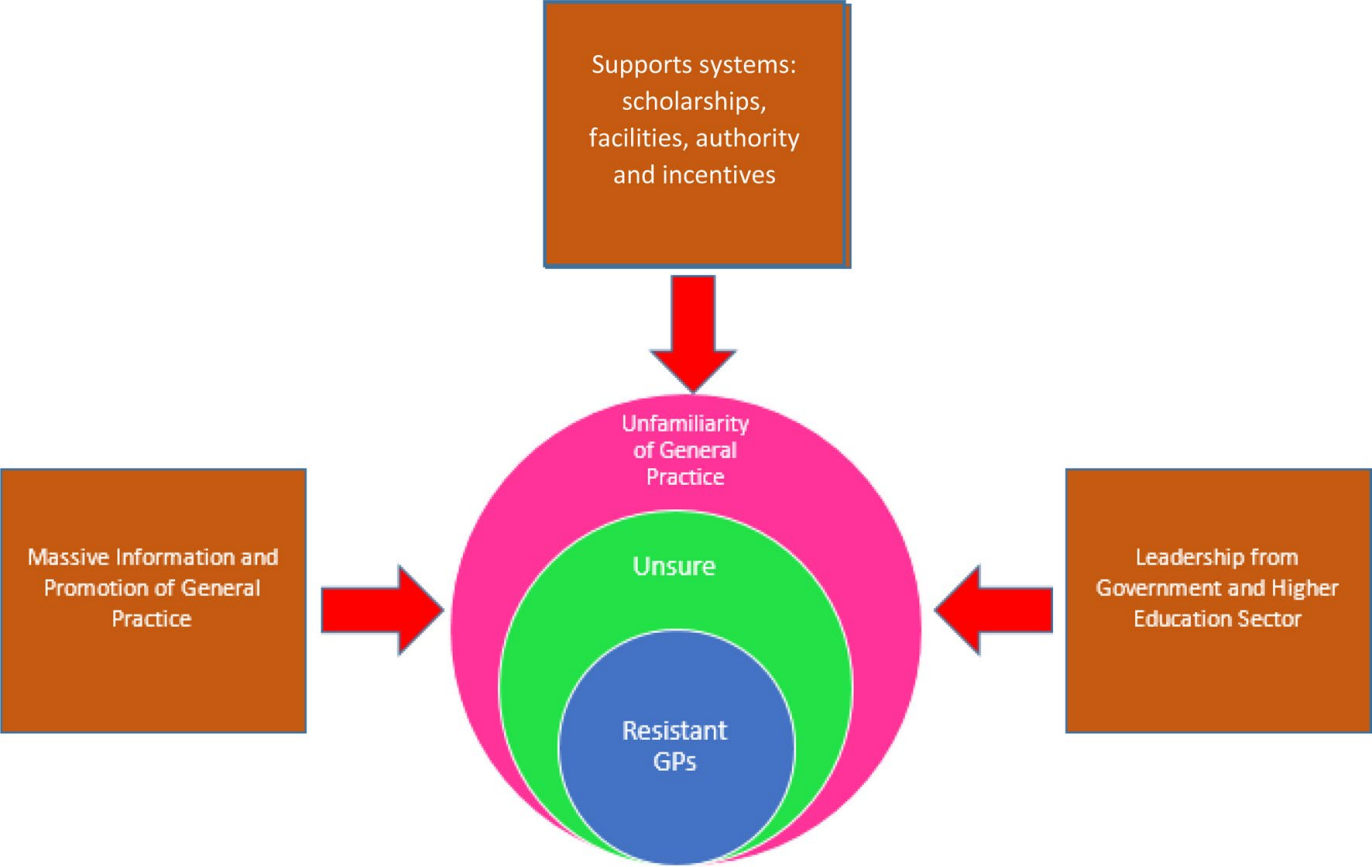
Practice supports, massive information dissemination, and leadership from both government and universities are potential to minimise the GPs’ resistance of PCP training in Indonesia

This phenomena of the Indonesian doctors’ reluctance to the PCP training is in line with an article from Haq, Ventres [23] that stated that family doctor programs in developing countries were lacking of recognition as a specialist training. In connection to this study finding, Indonesia is now left behind from other Asia Pacific countries for the establishment of family medicine as specialist training. The country still focused its primary care training materials in undergraduate level [24] and the current doctors have limited abstraction of family medicine as a unique specialisation. Therefore, massive considerations need to be taken by the Indonesian doctors and the government about the benefits of the developed primary care research and education, that the training could contribute to improve the health system as well as to provide a high quality health care for all people [14, 25] (Fig. 1).

As shown in the above findings of this research demonstrating that the current need for GP continuing education programs, the Indonesian government’s intention to upgrade the GPs quality with formal postgraduate training is currently on the appropriate decision [1, 14, 26–29]. In addition to offering annual postgraduate workshops, clear competence reviews and appropriate allocation of CPD credits for PCP courses/seminars/conferences are essential supports for the training program [30]. The leadership from the government sectors and extensive information dissemination are also needed to ensure the correct understanding of the GPs and to balance the resonance of the medical association resistance [15]. By these efforts, it is expected that the GPs can better understand the values of the PCP training and its impact on patients and the healthcare system [27, 28]. It is also strongly suggested that the government, GPs, and medical associations could create a plan together to minimize the tension and provide assistances for GPs who wish to take the PCP training.

Lastly, this research provided a foundation to further investigate the specialist doctors’ and other stakeholders’ views about Indonesian postgraduate training in primary care, to explore the challenges and any initiatives which may endorse the recruitment of PCP trainee in Indonesia.

## Strengths and limitations

This study was the first academic study to explore the Indonesian GPs views related to the PCP training. It was a small qualitative study with 19 GPs participants from Yogyakarta, Indonesia. The findings in this study should be appropriately interpreted and may not represent a nationwide range of doctors’ perspectives. However, the participants’ variation sampling strategy was able to achieve data collection from a range of sources, and the IPA analysis approach is valuable to strengthen the conclusions from the findings.
